# Day-3 *vs*. Day-5 fresh embryo transfer

**DOI:** 10.5935/1518-0557.20220027

**Published:** 2023

**Authors:** Putu Githa Garbhini, Anom Suardika, AAN Anantasika, IB Putra Adnyana, I Made Darmayasa, Nono Tondohusodo, Jaqueline Sudiman

**Affiliations:** 1 Obstetrics and Gynaecology Department, School of Medicine, Udayana University, Denpasar- Bali, Indonesia; 2 Bali Royal Hospital, Royal IVF clinic, Denpasar-Bali, Indonesia; 3 Anatomy Department, School of Medicine, Udayana University, Denpasar-Bali, Indonesia

**Keywords:** day-3 embryo transfer, day-5 embryo transfer, implantation rate, live birth rate, pregnancy rate

## Abstract

**Objective:**

Embryo transfer on day-5 has been associated with higher success rates, therefore our IVF clinics started to extend embryo culture until blastocyst stage. This study aimed to compare the success rates of day-3 vs. day-5 embryo transfers.

**Methods:**

We had 266 patients included, all having undergone ICSI, with 221 patients having undergone day-3 embryo transfers, and 45 patients having undergone day-5 embryo transfers. Patients with more than five good quality embryos on day-3 were chosen to prolong the culture of embryos into day-5.

**Results:**

There were no significant differences in patient characteristics, including baseline LH, FSH, Prolactin and Estradiol hormone levels. In addition, there were also no significant differences in rFSH total dosage and duration of stimulation day. Final estradiol levels, number of follicles, retrieved oocytes, matured oocytes, fertilized oocytes and number of embryos were significantly higher in day-5 compared to day-3 embryo transfer groups. Number of embryos transferred on day-3, were significantly higher compared to day-5. Neither group showed any significant differences in clinical pregnancy, implantation, multiple pregnancy or living birth rates. There were no differences in birth weights and lengths, head circumstances and Apgar Scores between both groups either in singleton or twin group.

**Conclusions:**

Transferring embryos at day-3 may provide the same benefits as day-5 embryo transfers to patients. However, more embryos were required to be transferred to achieve these comparable results.

## INTRODUCTION

Twelve to fifteen percent of reproductive couples suffer from infertility. Assisted reproductive technology (ART) is one of the advanced treatment options that can treat infertility problems. However, the success rate of ART has not improved significantly over the last 10 years. Pregnancy rate following ART is approximately 30-40%, with living birth rates per embryo transfer cycle of approximately 20-30% (ANZARD, 2018; De Geyter, 2019).

Several options have been proposed to increase the success rate of ART, such as prolonging embryo culture. Human embryos are usually transferred either on day-3 (cleavage stage) or day-5 (blastocyst stage). Prolonging embryo culture may increase the success rate of ART (Levron *et al.*, 2002; Gardner *et al.*, 1998; Schwärzler *et al.*, 2004). The reasons behind this new strategy are that embryos implant in the uterus at blastocyst stage naturally, and extended embryo culture until day-5 could lead to embryo selection that has demonstrated good outcomes (Gardner & Lane, 2003). Furthermore, embryos at blastocyst stage have their own genomic control and have already passed the maternal to zygotic transition (Johnson *et al.*, 2007; Braude *et al.*, 1988). Extended embryo culture until day-5 could also decrease the number of embryos being transferred, therefore reducing the incidence of multiple pregnancies (Gardner *et al.*, 2000; 2004). On the other hand, critics on extended embryo culture have reported an increase in embryo transfer cancellations due to a failure in embryos developing into blastocysts on the day of transfer, a failure to have extra embryos available to be frozen, the requirement of an excellent culture system, an increase in monozygotic twinning, and altered sex ratio in births (Johnson *et al.*, 2007; Ménézo *et al.*, 1999). Some studies have also reported that there were no differences in the success rates of IVF between embryo transfers on day 3 and embryo transfers on day 5 (Bungum *et al.*, 2003; Hatırnaz & Kanat Pektaş, 2017; Coskun *et al.*, 2000).

Due to the difficulty in cultivating embryos for a period of five days, it became common practice to transfer embryos at cleavage stage on days two or three. Until 2018, our IVF clinic only transferred embryos on day three; however, we sought to determine if extended embryo culture to day five could increase the success rate in our patients. Therefore, the aim of this study was to investigate pregnancy outcomes following day-3 and day-5 embryo transfers.

## MATERIALS AND METHODS

We conducted this retrospective cross-sectional study at the Royal IVF clinic, Bali Royal Hospital, Indonesia. We used secondary data from medical records of all patients who matched our criteria and had undergone intracytoplasmic sperm injection (ICSI) treatment between 2016 and 2019. This study was approved by the local ethics committee from Udayana University (667/UN14.2.2.VII.14/LT/2020).

We collected data from 266 patients, under the age of 40 years, submitted to ICSI. The inclusion criteria selected patients which were less than 40 years of age, normal responders with AFC ≥5 follicles, and had undergone a complete IVF program for which embryo transfers were performed on day three or day five.

There were 221 patients in the day-3 embryo transfer group and 45 patients in the day-5 embryo transfer group. All patients were administrated GnRH-antagonist protocols. Briefly, recombinant follicle-stimulating hormone (Gonal F, Serono, Switzerland) was used on day three of the menstrual cycle. The dosage of FSH was adjusted based on ovarian response and was assessed by transvaginal ultrasound and serum estradiol level. If two or more follicles reached a mean diameter ≥ 18 mm, the patients were injected with 10,000 IU of recombinant hCG (Serono, Switzerland). Ovum pick up was performed transvaginally, 34-38 hours after hCG injection. Two hours after oocyte retrieval the oocytes were denuded, and all matured oocytes were inseminated by ICSI. All fertilized oocytes were cultured for three days in culture media (G1, Vitrolife, Gothenburg, Sweden), or continued until day five using blastocyst media (G2, Vitrolife, Gothenburg, Sweden). In order to avoid the cancellation of embryo transfers, the clinic’s policy was to only choose patients showing at least five high-quality embryos on day three to extend embryo culture until day five. Embryos on day three were classified based on the grading by Kamardi *et al*. (2021). Grade one embryos were defined as 4-6 cells on day two, eight or more cells on day three, equal, fragmentation <10%, and no multinucleated blastomeres; grade-2 embryos were defined as 2-3 cells on day two, 6-7 cells on day three, equal or less equal, 10-20% fragmentation, and no multinucleated blastomeres; grade-3 embryos were defined as 0-2 cells on day two, 1-5 cells on day three, unequal, fragmentation >25%, with or without multinucleated blastomeres. Grade-1 and -2 embryos were considered top-quality embryos. Blastocysts were graded based on the modified grading by Richardson *et al.* (2015), which is defined by the size of the blastocele cavity (early, full, expanded); the size of inner cell mass; and trophectoderm layer distribution. Grade-A and - B blastocysts on day five were considered top-quality embryos. Early blastocysts or blastocysts with no inner cell mass were included among grade-C blastocysts. One to three embryos were transferred on day three or day five of embryo transfers.

The first parameters that we evaluated in this study were patient characteristics including age, BMI, number of cycles, type and duration of infertility, and baseline hormone levels (FSH, LH, prolactin and estradiol).

The second set of parameters that we measured were ovarian responses, including total rFSH dosage used, the length of ovulation induction, endometrial thickness on the hCG day, level of estradiol upon last evaluation, number of follicles, number of oocytes retrieved, number of matured oocytes, number of oocytes fertilized, number of embryos developed, and number of embryos transferred.

The third set of parameters that we measured were: chemical pregnancy (level of hCG ≥50 mIU/mL), clinical pregnancy (gestational sac seen on USG), the implantation rate (the ratio of gestational sac/number of embryos transferred), live birth rate (number of babies delivered/number of gestational sac), miscarriage and ectopic pregnancy rates.

The fourth set of parameters that we measured were neonatal outcomes, including birth weight, birth length, head circumstance, Apgar score and sex (table 4). 102 babies were born from day-3 embryo transfers, we collected data from 96 neonates born in our hospital. Out of 96 babies, 70 were singletons and 26 were twins. From 23 babies born from day-5 embryo transfers, we collected data from 22 neonates. Out of 22 babies, 16 were singletons and 6 were twins.

**Table 4:** Neonatal outcomes for day-3 vs. the day-5 embryo transfers.

	**Singletons**		**Twins**	
	**DAY-3**	**DAY-5**	**DAY-3**	**DAY-5**
Number of samples	76	16	20	6
Birth weight	3182.24±449.86^a^	3228.13±498.32^a^	2600.00±334.42^b^	2316.67±196.63^b^
Birth length	50.06±2.10	49.00±1.31	47.25±1.29	45.67±1.63
Head circumstances	34.13±1.39	34.19±1.42	32.20±1.61	33.17±0.52
Apgar score ≥ 7 <7	76 0	16 0	19 1	6 0
Sex Female Male	39 37	8 8	9 11	3 3

Different superscripts denote statistical difference (*p*<0.01).

### Statistical analysis

The data were expressed as mean ± SD or number (percentage) and analyzed using the SPSS version 25.0. The data were analyzed using the independent t-test from difference subjects. Due to small samples in the day-5 compared to the day-3 embryo transfer groups, we used a non-parametric test (Mann-Whitney test) to compare the means/medians. Proportions were compared using the Chi-square test. All statistical significance was set as *p*<0.05.

## RESULTS

### Baseline Characteristic

Table 1 depicts baseline characteristics of IVF embryo transfer patients from days -3 and -5 groups. There were no differences in age, BMI, infertility type, number of cycles, type of infertility and duration of infertility. There were no significant differences in baseline FSH, LH, Prolactin and Estradiol levels in both groups.

**Table 1:** Baseline characteristics for day-3 vs. day-5 embryo transfers.

**Characteristic**	**Day-3 embryo transfer**	**Day- 5 embryo transfer**
Number of sample	221	45
Age (years)	32.2±4.4	31.2±3.9
BMI (kg/m^2^)	23.2±2.8	24.1±2.9
Number of cycles (%) 1 >1	188 (85.1%) 33 (14.9%)	39 (86.7%) 6 (13.3%)
Type infertility Primary Infertility	163 (73.8%) 58 (26.2%)	27 (60%) 18 (40%)
Duration of infertility (years)	5.4±3.7	4.1±3.0
Basal Hormonal levels FSH (mIU/mL) LH (mIU/mL) Prolactin (ng/mL) Estradiol (pg/mL)	5.7±2.1 3.1±1.3 22.4±10.3 35.3±10.8	5.2±1.5 3.1±1.4 24.7±15.0 37.4±10.4

### Ovarian stimulation parameters and IVF results

Table 2 shows that the two groups were comparable with regards to rFSH dose and duration of stimulation. There were significant differences in final estradiol level, number of follicles, number of retrieved oocytes, number of mature oocytes, and number of fertilized oocytes, number of high-quality embryos on day three in the day-5 embryo transfer group, compared to the day-3 embryo transfer group (*p*<0.01). There were significantly higher numbers of embryos transferred in the day three compared to the day-5 embryo transfer groups.

**Table 2:** Ovarian response and embryo development for day-3 vs. day-5 embryo transfers.

**Characteristic**	**Day-3 embryo transfer**	**Day-5 embryo transfer**
Total dosage of rFSH (IU)	1665.0±557.5	1836.6±545.8
Duration of stimulation (Days)	7.8±1	7.9±1.1
Final estradiol level (pg/mL)	2054.9±766.1^a^	2877.5±1331.2^b^
Number of follicles	14.0±7.7^a^	24.2±9.9^b^
Number of retrieved oocytes	8.5±4.2^a^	13.5±5.8^b^
Number of mature oocytes	6.6±3.5^a^	10.4±4.0^b^
Number of fertilized oocytes	5.1±2.9^a^	8.6±3.7^b^
Number of embryos on day-3	5.0±2.8^a^ (98%)	8.4±3.7b (99.2%)
(% fertilized oocytes)		
Grade-1 embryo	2.1±2.0^a^ (40.3%)	5.0±2.4^b^ (56.2%)
Grade-2 embryo	1.8±1.8^a^ (35.4%)	3.0±2.8^b^ (30.2%)
Grade-3 embryo	1.2±1.3 (22.3%)	1.6±2.0 (12.9%)
Number of blastocyst on day 5	N/A	5.2±2.8 (73%)
(% of day-3 embryos)		
Grade A	N/A	0.4±0.7 (4.7%)
Grade B	N/A	2.6±1.9 (29.6%)
Grade C	N/A	3.4±2.2 (38.7%)
Number of embryo transfer	2.3±0.7^a^	2.1±0.4^b^
Endometrial thickness (mm)	10.7±2.2	10.6±2.6

Different superscripts denote statistical difference (*p*<0.01).

### Pregnancy outcomes

Looking at pregnancy outcomes, there were no statistically significant differences in chemical pregnancy, clinical pregnancy, implantation, live delivery or multiple pregnancy rates between the groups (Table 3). Two out of 93 pregnant patients in the day three embryo transfer group had miscarriages and three had ectopic pregnancies. We only found one patient in the day-5 embryo transfer group who suffered from miscarriage.

**Table 3:** ICSI outcomes for day-3 vs. day-5 embryo transfers.

**Characteristic**	**Day-3 embryo transfer**	**Day-5 embryo transfer**
Chemical pregnancy rate (%)	94/221 (42.5%)	21/45 (46.7%)
Clinical pregnancy rate (%)	93/221 (42.1%)	21/45 (46.7%)
Implantation rate (%)	134/511 (26.2%)	31/94 (33%)
Multiple pregnancy (%)	36/221 (16.3%)	9/45 (20%)
Live birth rate (%)	102/134 (76.1%)	23/31 (74.2%)
Miscarriage (%)a	2/93 (2.2%)	1/21 (4.8%)
Ectopic pregnancy (%)a	3/93 (3.2%)	0

a= the number is too small for statistical analysis.

### Neonatal Outcomes

There were no significant differences in birth weights, birth lengths, head circumstances infants between day-3 and day-5 embryo transfers in either the singletons or the twins group. Only one baby in the day-3 transfer group had lower Apgar score. Day-3 and day-5 embryo transfers had almost the same sex ratios.

## DISCUSSION

IVF success depends on endometrium receptivity and embryo quality. In recent years, advances in embryo culture systems and media development have encouraged clinics to extend embryo transfer until the blastocyst stage. The advantages of blastocyst transfer (day five to six) compared to cleavage stage transfer (day two to three) are still controversial. Some reports suggest that blastocyst transfer is more favorable than cleavage transfer (Levron *et al.*, 2002; Gardner *et al.*, 1998; Schwärzler *et al.*, 2004; Papanikolaou *et al.*, 2005; Karaki *et al.*, 2002). However, other reports indicate that there are no differences in success rates between blastocyst and cleavage transfers (Bungum *et al.*, 2003; Hatırnaz & Kanat Pektaş, 2017; Coskun *et al.*, 2000; Alfaraj *et al.*, 2017). Results of Cochrane studies, that examine day-3 compared to day-5 embryo transfer, are also variable and inconsistent (Glujovsky *et al.*, 2012; 2016). A Cochrane study that included 27 randomized controlled trials suggested that blastocyst transfer may increase pregnancy rates between 3-10%, and live birth rates between 3-13%, compared to cleavage-stage transfers (Glujovsky *et al.*, 2016). However, cumulative clinical pregnancy rates from cleavage stage, derived from fresh and frozen cycles, resulted in the same or slightly better results compared to blastocyst transfers (Glujovsky *et al.*, 2012; 2016). This may be due to an increase in embryo cancellation and decrease in the number of embryos frozen in the blastocyst group. In our data, to avoid embryo cancellation, we only chose patients with more than five good quality embryos on day three, to extend embryo culture to blastocyst stage and therefore not have embryo transfer cancellation data to report. A prospective randomized controlled trial also used the same strategy as us, in which poor responders or low number of high-quality embryos on day three had embryo transfer on days two or three (Hatırnaz & Kanat Pektaş, 2017). They found no statistically significant differences in implantation, clinical pregnancies, miscarriages, perinatal death, and live birth rates between days -3 and -5 transfer groups. Similar results were found in this present study, where there were no significant differences in pregnancies, implantation, live birth, and multiple pregnancies between the groups. However, to achieve these comparable results, more embryos were required to be transferred in the day-3 group compared to day-5 embryo transfer groups. A study by Schwärzler *et al.* (2004), using patients with at least one previous implantation failure with higher follicle number, found that day-5 transfers were significantly superior in terms of pregnancy and live birth rates when compared to day three transfers. They also found more multiple gestations among patients following day-5 transfers, even though the number of embryos transferred was similar to that of day-3 transfers. This was probably due to a lower rate of aneuploidy at blastocyst stage compared to the cleavage stage (Staessen *et al.*, 2004).

Culturing embryos until the blastocyst stage also requires an excellent culture system. In this study, around 73% of cleavage embryos on day three developed into blastocyst stage on day five. From 73% blastocyst rate, around 34.3% became grade A and grade B blastocysts. The number of blastocysts derived from cleavage embryos is different from one unit to another and depends on the quality of oocytes, sperm, and underlying infertility disease. In general, the blastocyst rate from cleavage stage ranges from 28% to 60.3% (Glujovsky *et al.*, 2012; 2016). A prospective randomized study indicated that single blastocyst transfers from young-age women with excellent quality had significantly higher pregnancy rates compared to single embryo cultures on day three (40.8% and 25.6%, respectively). However, this number drops from 40.8% to 26.2% if there is only one moderate quality blastocyst available on the day of embryo transfer (Zech *et al.*, 2007). It appears that blastocyst transfers are more favorable if it is aimed at transferring only a single embryo.

Looking at miscarriage and ectopic pregnancy rates, there were no significant differences in both groups; however, our sample was too small to be statistically analyzed. Meta-analysis studies have shown no significant differences in miscarriage rates (Glujovsky *et al.*, 2012; 2016). A study from Schwärzler *et al.* (2004) also reported that there were no differences in genetic birth defects between the groups.

This present study shows that there are no significant differences in birth weights between day-3 *vs*. day-5 embryo transfer groups. Similar results have been reported, that there were no differences in mean birth weights in singleton groups between fresh embryo transfer on day-3 and day-5 groups (Rahil *et al.*, 2018). However, it has been shown that frozen embryo transfer on day five was associated with a lower birthweight compared to frozen embryo transfer on day three (De Vos *et al.*, 2018). On the contrary, another study with large samples has shown that there was a significantly higher birth weight after good- quality blastocyst transfer on day five or day six compared to day-3 embryo transfers (Wang *et al.*, 2021). Multiple births are the complication associated with multiple embryos transferred, associated with adverse health outcomes, including low birth weight. In this present study, the birth weights in the twins’ group in either day-3 or day-5 groups are significantly lower compared to singleton groups; however, there were no genetic abnormalities in the twins’ groups. Only one baby had Apgar score below 7.

To conclude, transferring embryos on day three may provide the same benefits as day-5 embryo transfers to patients; however, more embryos may require to be transferred. Despite this, our results also do not support the notion that embryos from all patients showed an equal implantation potential on days 3 or 5. Larger data samples are required for future studies, as well as reports of total pregnancy rates, which include frozen embryo transfers.

## References

[r1] Alfaraj S, Alzaher F, Alshwaiaer S, Ahmed A (2017). Pregnancy outcome of day three versus day five embryo transfer: A
retrospective analysis. Asian Pac J Reprod.

[r2] ANZARD - Australian and New Zealand Assisted Reproduction
Database Assisted Reproduction in Australia and New Zealand 2018.

[r3] Braude P, Bolton V, Moore S (1988). Human gene expression first occurs between the four- and
eight-cell stages of preimplantation development. Nature.

[r4] Bungum M, Bungum L, Humaidan P, Yding Andersen C (2003). Day 3 versus day 5 embryo transfer: a prospective randomized
study. Reprod Biomed Online.

[r5] Coskun S, Hollanders J, Al-Hassan S, Al-Sufyan H, Al-Mayman H, Jaroudi K (2000). Day 5 versus day 3 embryo transfer: a controlled randomized
trial. Hum Reprod.

[r6] De Geyter C, ESHRE Meeting 2019 European pregnancy rates from IVF and ICSI ‘appear to have reached a
peak’.

[r7] De Vos A, Santos-Ribeiro S, Van Landuyt L, Van de Velde H, Tournaye H, Verheyen G (2018). Birthweight of singletons born after cleavage-stage or blastocyst
transfer in fresh and warming cycles. Hum Reprod.

[r8] Gardner DK, Schoolcraft WB, Wagley L, Schlenker T, Stevens J, Hesla J (1998). A prospective randomized trial of blastocyst culture and transfer
in in-vitro fertilization. Hum Reprod.

[r9] Gardner DK, Lane M, Stevens J, Schlenker T, Schoolcraft WB (2000). Blastocyst score affects implantation and pregnancy outcome:
towards a single blastocyst transfer. Fertil Steril.

[r10] Gardner DK, Lane M (2003). Blastocyst transfer. Clin Obstet Gynecol.

[r11] Gardner DK, Surrey E, Minjarez D, Leitz A, Stevens J, Schoolcraft WB (2004). Single blastocyst transfer: a prospective randomized
trial. Fertil Steril.

[r12] Glujovsky D, Blake D, Farquhar C, Bardach A (2012). Cleavage stage versus blastocyst stage embryo transfer in
assisted reproductive technology. Cochrane Database Syst Rev.

[r13] Glujovsky D, Farquhar C, Quinteiro Retamar AM, Alvarez Sedo CR, Blake D (2016). Cleavage stage versus blastocyst stage embryo transfer in
assisted reproductive technology. Cochrane Database Syst Rev.

[r14] Hatırnaz Ş, Kanat Pektaş M (2017). Day 3 embryo transfer versus day 5 blastocyst transfers: A
prospective randomized controlled trial. Turk J Obstet Gynecol.

[r15] Johnson N, Blake D, Farquhar C (2007). Blastocyst or cleavage-stage embryo transfer?. Best Pract Res Clin Obstet Gynaecol.

[r16] Kamardi S, Surya IHW, Mahendra INB, Adnyana IP, Suardika A, Tondohusodo N, Sudiman J (2021). Impact of body mass index on intracytoplasmic sperm injection in
women with polycystic ovary syndrome. Zygote.

[r17] Karaki RZ, Samarraie SS, Younis NA, Lahloub TM, Ibrahim MH (2002). Blastocyst culture and transfer: a step toward improved in vitro
fertilization outcome. Fertil Steril.

[r18] Levron J, Shulman A, Bider D, Seidman D, Levin T, Dor J (2002). A prospective randomized study comparing day 3 with
blastocyst-stage embryo transfer. Fertil Steril.

[r19] Ménézo YJ, Chouteau J, Torelló J, Girard A, Veiga A (1999). Birth weight and sex ratio after transfer at the blastocyst stage
in humans. Fertil Steril.

[r20] Papanikolaou EG, D’haeseleer E, Verheyen G, Van de Velde H, Camus M, Van Steirteghem A, Devroey P, Tournaye H (2005). Live birth rate is significantly higher after blastocyst transfer
than after cleavage-stage embryo transfer when at least four embryos are
available on day 3 of embryo culture. A randomized prospective study. Hum Reprod.

[r21] Rahil T, Ashcraft L, Rastegar V, Sites CK, Schoen C (2018). Pregnancy and perinatal outcomes after fresh blastocyst (D5) and
cleavage (D3) stage elective single embryo transfer (ESET)
cycles. Fertil Steril.

[r22] Richardson A, Brearley S, Ahitan S, Chamberlain S, Davey T, Zujovic L, Hopkisson J, Campbell B, Raine-Fenning N (2015). A clinically useful simplified blastocyst grading
system. Reprod Biomed Online.

[r23] Schwärzler P, Zech H, Auer M, Pfau K, Göbel G, Vanderzwalmen P, Zech N (2004). Pregnancy outcome after blastocyst transfer as compared to early
cleavage stage embryo transfer. Hum Reprod.

[r24] Staessen C, Platteau P, Van Assche E, Michiels A, Tournaye H, Camus M, Devroey P, Liebaers I, Van Steirteghem A (2004). Comparison of blastocyst transfer with or without preimplantation
genetic diagnosis for aneuploidy screening in couples with advanced maternal
age: a prospective randomized controlled trial. Hum Reprod.

[r25] Wang N, Zhao X, Ma M, Zhu Q, Wang Y (2021). Effect of Day 3 and Day 5/6 Embryo Quality on the Reproductive
Outcomes in the Single Vitrified Embryo Transfer Cycles. Front Endocrinol (Lausanne).

[r26] Zech NH, Lejeune B, Puissant F, Vanderzwalmen S, Zech H, Vanderzwalmen (2007). Prospective evaluation of the optimal time for selecting a single
embryo for transfer: day 3 versus day 5. Fertil Steril.

